# Prevalence and Distribution of Avian Influenza Viruses in Domestic Ducks at the Waterfowl-Chicken Interface in Wetlands

**DOI:** 10.3390/pathogens9110953

**Published:** 2020-11-16

**Authors:** Mohammad M. Hassan, Ariful Islam, Rubyath B. Hasan, Md. K. Rahman, Richard J. Webby, Md. A. Hoque, Mohamed E. El Zowalaty

**Affiliations:** 1 Faculty of Veterinary Medicine, Chattogram Veterinary and Animal Sciences University, Chattogram 4225, Bangladesh; arif@ecohealthalliance.org (A.I.); rubyathhasan.official@gmail.com (R.B.H.); kaisarrahman@ecohealthalliance.org (M.K.R.); md.hoque@my.jcu.edu.au (M.A.H.); 2 Centre for Integrative Ecology, School of Life and Environmental Science, Deakin University, Geelong Campus, VIC 3216, Australia; 3 EcoHealth Alliance, New York, NY 10001-2023, USA; 4 Division of Virology, Department of Infectious Diseases, St. Jude Children’s Research Hospital, Memphis, TN 38105, USA; richard.webby@stjude.org; 5 Department of Clinical Sciences, College of Medicine, University of Sharjah, Sharjah 27272, UAE; 6 Zoonosis Science Center, Department of Medical Biochemistry and Microbiology, Uppsala University, SE-75 123 Uppsala, Sweden

**Keywords:** avian influenza, prevalence, risk factors, real-time reverse-transcription-polymerase chain reaction, c-ELISA, waterfowl, ducks, interface, wetlands, Bangladesh

## Abstract

Ducks are a natural reservoir of influenza A viruses (IAVs) and can act as a reassortment vessel. Wetlands, such as Hakaluki and Tanguar haor in Bangladesh, have unique ecosystems including domestic duck (*Anas platyrhynchos domesticus*) rearing, especially household and free-range ducks. A cross-sectional study was, therefore, conducted to explore avian influenza status and its distribution and risk factors in the wetland areas. During the three consecutive winters of 2015–2017, specifically in December of these years, we collected a total of 947 samples including blood, oropharyngeal and cloacal swabs from domestic ducks (such as free-range ducks (*n* = 312 samples) and household ducks (*n* = 635 samples) in wetlands. We screened serum samples using a nucleoprotein competitive enzyme-linked immunosorbent assay (c-ELISA) to estimate seroprevalence of IAV antibodies and swab samples by real-time reverse transcriptase polymerase chain reaction (rRT-PCR) to detect IA viral M gene. Eleven M gene positive samples were subjected to sequencing and phylogenetic analysis. Serological and viral prevalence rates of IAVs were 63.8% (95% CI: 60.6–66.8) and 10.7% (8.8–12.8), respectively. Serological and viral RNA prevalence rates were 51.8% (95% CI: 47.2–56.4) and 10.2% (7.6–13.3) in Hakaluki haor, 75.6% (71.5–79.4) and 11.1% (8.5–14.3) in Tanguar haor, 66.3% (62.5–69.9) and 11.2% (8.8–13.9) in household ducks and 58.7% (52.9–64.2) and 9.6% (6.5–13.4) in free-range ducks, respectively. The risk factors identified for higher odds of AI seropositive ducks were location (OR = 2.9, 95% CI: 2.2–3.8, *p* < 0.001; Tanguar haor vs. Hakaluki haor), duck-rearing system (OR = 1.4, 1.1–1.8, household vs. free-range), farmer’s education status (OR = 1.5, 1.2–2.0, *p* < 0.05 illiterate vs. literate) and contact type (OR = 3.0, 2.1–4.3, *p* < 0.001; contact with chicken vs. no contact with chicken). The risk factors identified for higher odds of AI viral RNA positive ducks were farmer’s education status (OR = 1.5, 1.0–2.3, *p* < 0.05 for illiterate vs literate), contact type (OR = 2.7, 1.7–4.2, *p* < 0.001; ducks having contact with chicken vs. ducks having contact with waterfowl). The phylogenetic analysis of 11 partial M gene sequences suggested that the M gene sequences detected in free-range duck were very similar to each other and were closely related to the M gene sequences of previously reported highly pathogenic avian influenza (HPAI) and low pathogenic avian influenza (LPAI) subtypes in waterfowl in Bangladesh and Southeast Asian countries. Results of the current study will help provide significant information for future surveillance programs and model IAV infection to predict the spread of the viruses among migratory waterfowl, free-range ducks and domestic poultry in Bangladesh.

## 1. Introduction

Avian influenza (AI) is an infectious disease caused by influenza A viruses (IAVs) in the family *Orthomyxoviridae*, which are negative-sense, single-stranded RNA viruses. Bird-to-bird transmission is the general form of disease maintenance for AI [1], although viruses infrequently cross the species barrier and can infect humans and many other animals [2]. In domestic poultry, avian IAVs can be classified as highly pathogenic avian influenza (HPAI) or low pathogenic avian influenza (LPAI) based on lethality for chicken. Waterfowl, including ducks, are the main natural reservoir and source for all subtypes of avian influenza viruses [3]. Ducks may act as a reassortment vessel for IAVs [4,5]. During winter, open wetlands in Bangladesh are shared by large numbers of migratory waterfowl, resident birds and household ducks. These agro-ecological landscapes act as important sites for interaction of migratory waterfowl, household ducks and chickens, amplifying the risk of cross-infection of IAVs [6,7]. Free-range ducks and semi-scavenging household ducks can serve as bridging hosts and reassortment vessels for new influenza virus subtypes between wild waterfowl, domesticated birds and humans [8,9]. HPAI H5N1 of clade 2.3.2.1 viruses were isolated from wild waterfowl in 2010 and were reported to be circulating among poultry [10]. Previous studies suggested that the virus may have been introduced to Bangladesh through migratory birds [10,11]. Clades of HPAI viruses can evolve over time by the process of antigenic shift and drift in infected ducks that may not show any visible signs of illness [12].

In earlier published data, AI seroprevalence in household and free-range ducks in various regions of Bangladesh was found to be 30–90.2% [8,9,13,14]. AI viral prevalence in the same populations was reported to be 24–89%, irrespective of subtype [15,16]. HPAI H5N1 subtype was reported in ducks in live bird markets of Bangladesh since 2007 [11]. The country does not have a formal health information system for duck rearing [13] and very few studies have explored the risk factors associated with IAVs in Bangladesh [17,18,19]. Notably, no data are available about the investigations of AI serological and viral prevalence with associated risk factors in ducks in different rearing systems in Haor areas of Bangladesh. Haor (back swamp) is a bowl or saucer-shaped depression between the natural levees of a river and covers about one-fourth of the northeastern region of Bangladesh [20]. For people living in these areas, duck farming is one of the most important means of livelihood and a substantial tool for women’s empowerment [21] because it can contribute to food security directly in terms of nutrient values and it economically enables rural women, which passively influences their selective spending on food and healthcare [22]. The Ramsar Convention on Wetlands acknowledged Tanguar and Hakaluki haors as key international sites for conservation and sustainable use, given their large waterfowl (resident and exotic) populations and exclusive ecosystem. These areas intersect with the route of the Central Asian flyway of migratory waterfowl and provide roosting, breeding and feeding resources for waterfowl [23]. Therefore, these areas create a unique ecology to study IAVs. In Bangladesh, ducks are mostly reared in free-range (nomadic) and household rearing systems. Household ducks are kept overnight near or within the farmer’s house and travel only over short distances [24]. The major challenges for raising household ducks are infectious diseases, husbandry training gaps, financial constraints, poor veterinary services and a disorganized marketing system [25,26]. In contrast, free-range ducks scavenge in a large flock (100–1000) in post-harvest paddy fields and water bodies during the day and stay in temporary enclosures overnight. The large water bodies serve as a site for intermingling between free-range ducks and wild waterfowl populations, resulting in the transmission of IAVs [27]. For the farmers’ families, it is a common practice to share dwelling places with ducks and chickens [13] and such close proximity between humans and avian species may results in the emergence of novel IAV strains of pandemic potential circulating between humans, ducks and chickens.

The precursor strain (A/goose/Guangdong/1/1996) of the currently circulating HPAI H5 subtype first emerged in waterfowl in China in 1996 [28]. HPAI H5N1 subtype was reported in domestic poultry in Bangladesh for the first time in 2007 [11]. Between 2007 and 2020, eight cases of influenza A H5N1 subtype including one fatality were reported in humans in Bangladesh [29]. The outbreak caused the country to face devastating economic losses of an estimated US$746 million in the poultry sector as well as adverse social effects [30]. Therefore, it is necessary to establish the first line of defense against AI through continuous monitoring of emerging viruses at targeted sentinel sites to allow for better preparedness, early detection and rapid response. The present study was carried out to study IAVs in domestic ducks in wetland areas in Sylhet in Bangladesh and aimed to determine the serological and virological prevalence of IAVs with potential risk factors in different rearing systems, the temporal and spatial distribution, and the phylogenetic relatedness of avian influenza viruses in the duck population.

## 2. Materials and Methods

### 2.1. Ethical Approval

The study protocol was approved by the Chattogram Veterinary and Animal Sciences University Ethics Committee (reference number: CVASU/Dir (Research and Extension) AEEC/2015/02), Bangladesh. Individual written consents were obtained from participants prior to the interview. Sampling procedure was followed to minimize animal suffering throughout the study.

### 2.2. Study Locations

Two significant marsh wetlands, namely Hakaluki and Tanguar haors of northeastern Bangladesh, were chosen as the study sites because these wetlands intake millions of migratory birds [31]. Hakaluki haor is the largest haor in Bangladesh and partly extends in Sylhet and Moulvibazar districts. It lies between 24°35′ N to 24°44′ N and 92°00′ E to 92°08′ E. The wetland elevation of Hakaluki haor is below 9 m [32]. Tanguar haor is located in the Sunamganj district and lies between 25°09′ N to 25°12′ N and 91°04′ E to 91°07′ E, covering an approximate area of 10,000 ha. The haor is 2.5–5.5 m above sea level. Both of the haors are just below the hilly regions of the states of Assam, Meghalaya and Tripura of India and are most affected by the rainfall pattern of the upstream catchments [33]. The total human population of the seven haor districts is approximately 20 million who share different ethnic population percentage mainly in the surrounding highland of Habiganj and Moulvibazar. Despite the economic importance of the region, humans in the haor areas are more impoverished than people in other parts of the country due to landlessness, mono-crop cultivation, seasonal unemployment and natural calamities. The main economic activity is agriculture and duck rearing is considered an alternative activity of livelihood next to fishing in haor areas [21].

**Figure 1:**
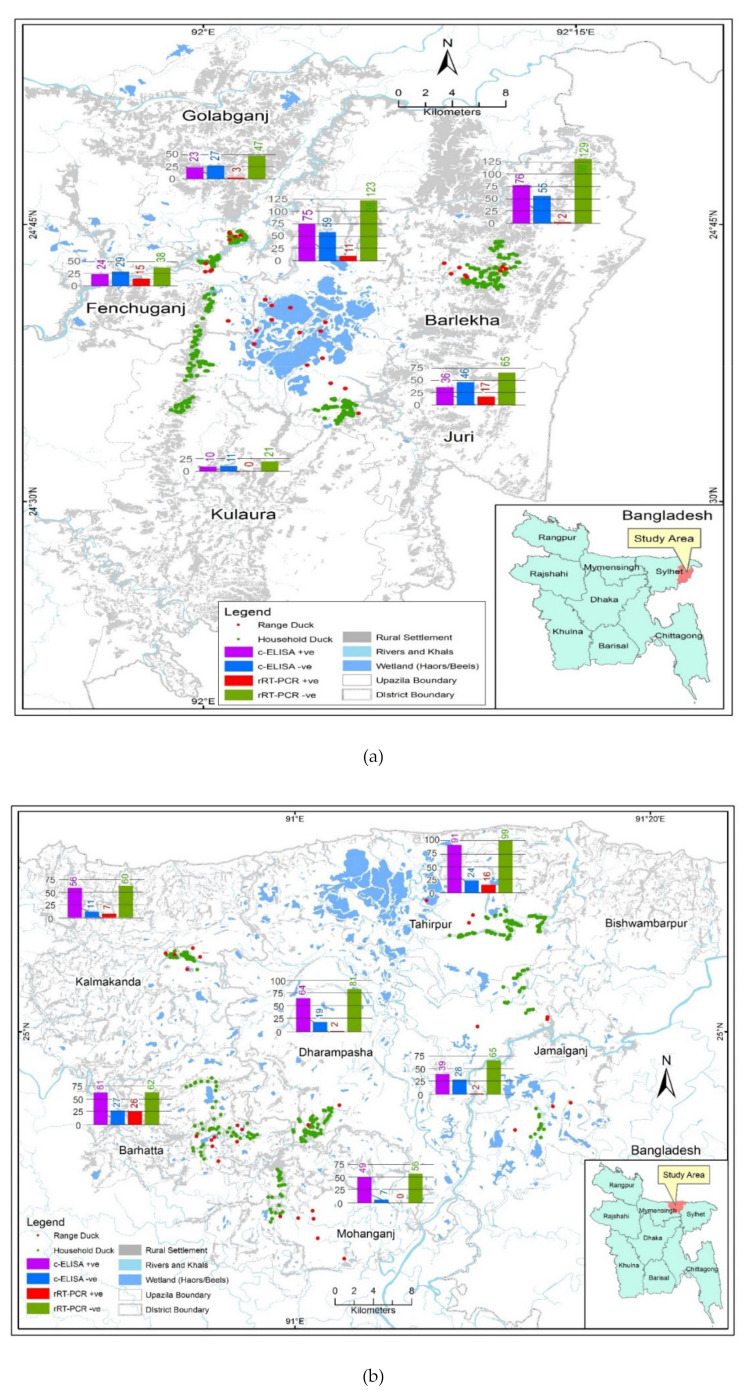
Distribution of avian influenza (AI) serological and matrix gene positive and negative numbers detected in free-range and household ducks in the (**a**) Hakaluki haor area and (**b**) Tanguar haor area in Bangladesh between 2015–2017. (results were presented in bar diagrams; violet colored bars indicated competitive enzyme-linked immunosorbent assay (c-ELISA) positive samples, blue colored bars indicated c-ELISA negative samples, red colored bars indicated real-time reverse transcriptase polymerase chain reaction (rRT-PCR) positive samples, and green colored bars indicated rRT-PCR negative samples).

A Geographic Information System (GIS) program (ArcGIS-ArcMap version 10.2; Environmental System Research Institute, Redlands, CA, USA) was used to prepare a map showing the study sites. Administrative and wetland shapefiles with haor positioning for Bangladesh (Figure 1a,b) were obtained from Humanitarian Data Exchange v1/1.43.6, the United Nations Office for the Coordination of Humanitarian Affairs (Available at https://data.humdata.org).

### 2.3. Study Design

Three cross-sectional studies were carried out to determine the prevalence and distribution of IAVs in free-range (nomadic) and household ducks in Hakaluki and Tanguar haors of Bangladesh from 2015 to 2017. During the three subsequent years, the month of December was identified as the time point for collecting the samples representing the winter season when the density of migratory birds remained at maximum levels.

### 2.4. Source Population and Sampling

Ducks belonging to free-range flocks and household flocks of the study sub-district were considered as the reference population. Individual apparently healthy ducks of any age and sex were registered at the sampling units for the study. Household duck flocks or ducks raised in households scavenge in premises in close proximity to small waterbodies without access to major wetlands. Free-range flocks consist of free-range ducks which are kept unattended for most of their life after being released in wetlands at four weeks of age. They mingle freely with migratory wild birds and are rounded-up for sale approximately after 48 weeks. Sample collection was performed from duck flocks owned by farmers who participated in the study. For each year, we targeted a group of at least 100 ducks based on types of contacts with ducks in their habitat. Contact types were categorized into: (i) ducks sharing a habitat with only household ducks, (ii) ducks sharing a habitat with both household ducks and chickens (*Gallus gallus domesticus*) or (iii) ducks having contact with wild birds such as Common moorhen (*Gallinula chloropus*), Brown-headed gull (*Chroicocephalus brunnicephalus*), and Black drongo (*Dicrurus macrocercus*) or migratory waterfowls such as Tufted duck (*Aythya fuligula*), Northern pintail (*Anas acuta*), and Lesser whistling duck (*Dendrocygna javanica*). The inclusion criterion for sampling at the household level was a single household having a minimum number of five ducks. We randomly sampled one duck from each household. In case of free-range ducks, we randomly sampled five ducks from a flock with a size of 300–1000 ducks. For both free-range and household farms, ducks were randomly selected during sampling.

### 2.5. Sample Collection

Cloacal swabs, oro-pharyngeal swabs, and blood samples were collected from each duck. Each of the cloacal and oropharyngeal swab samples was placed separately into a vial containing 1 mL of sterile viral transport media as previously described [34]. The samples were immediately placed in liquid nitrogen tanks and then kept in the laboratory at −80 °C after transport.

Whole-blood samples (0.5–3 mL, in all cases <1% of body weight) were drawn aseptically from the wing veins or jugular veins of the duck and samples were immediately transferred to 3 mL vacutainer tubes (BD Vacutainer, Franklin Lakes, NJ, USA) and were individually labelled for identification. Blood samples were subsequently allowed to clot at ambient temperature and kept refrigerated overnight, followed by centrifugation at 10,000 rpm for 30 min at 4 °C to separate serum. Serum samples were then transferred into cryovials and kept at −20 °C for further testing for the determination of IAV antibody.

### 2.6. Data Collection

For obtaining epidemiological information, a structured questionnaire was deployed during sample collection. The questionnaire was provided to all sampling units with a unique identifier number incorporated with each sample. Farmers’ demographic data including education level were collected. At the same time, farm data were collected on the location (Hakaluki/Tanguar haor area), duck rearing system (household/free-range) and contact type for ducks in different rearing systems (Supplementary Table S1).

### 2.7. Competitive Enzyme-Linked Immunosorbent Assay (c-ELISA)

Serum samples were tested using competitive enzyme-linked immunosorbent assay (c-ELISA) according to the Australian Animal Health Laboratory (Geelong, Melbourne, Victoria, Australia) protocol [35]. In c-ELISA, avian influenza antibodies were detected against IAV nucleoprotein (NP) using commercial test kit ID.vet ID Screen^®^ (Catalog No. FLUACAver 1216 GB; Sensitivity 100% and Specificity 96%; ID.vet, Garbles, France). The results were interpreted according to the manufacturer’s guidelines and signal-to-noise ratio (the quotient of the sample mean absorbance divided by negative control mean absorbance) of 0.45 or less was considered to be positive. A blood sample was collected from each duck and the result of the c-ELISA of each sample was indicated at individual duck level as positive or negative.

### 2.8. RNA Extraction and rRT-PCR

Influenza A viral RNA was extracted from individual cloacal and oropharyngeal samples (*n* = 947) using the MagMAX^TM^-96 Viral RNA Isolation Kit (Thermo Fisher Scientific, Waltham, MA, USA) according to the manufacturer’s guidelines. The AgPath-ID One-Step real-time reverse transcriptase polymerase chain reaction (rRT-PCR) kit (Thermo Fisher Scientific, MA, USA) was used for rRT-PCR testing. RNA extracts were tested individually for the presence of IAV RNA by rRT-PCR (Sensitivity: 99.5% and Specificity: 88.2%) using influenza A virus matrix (M) gene specific primers and probe. Samples were screened for of the presence of IAV) RNA, using matrix-gene specific real-time reverse transcription-polymerase chain reaction (rRT-PCR) using primers and probe as previously described [36]. The primers and probe sequences for M gene were as follows: 5′-AGATGAGTCTTCTAACCGAGGTCG–3′ (M+25), 5′-TGCAAAAACATCTTCAAGTCTCTG–3′ (M-124) and 5′-FAM-TCAGGCCCCCTCAAAGCCGA-TAMRA-3′ (M + 64). Samples were considered positive when the cycle threshold value (Ct) was less than 40 as previously described [37].

### 2.9. Sequencing and Phylogenetic Analysis

RNA was extracted from all M-gene positive samples using Trizol method (Invitrogen, Carlsbad, CA, USA) and Direct-Zol RNA MiniPrep Kit (Zymo Research, Irvine, CA, USA) according to the manufacturers’ guidelines. cDNA was synthesized using Superscript III (Invitrogen, Carlsbad, CA, USA) according to the manufacturer’s instructions. RT-PCR was performed for the amplification of IAV M-gene using primers FLUAV-MU44(GTCTTCTAACCGAGGTCGAAACG) and FLUAV-M-L287 (GCATTTTGGACAAAGCGTCTACG) to produce a 243-bp amplicon as previously described [38]. PCR products were purified using the Exo SAP-IT^®^ PCR product clean-up kit (Affymetrix, Ohio, USA) manufacturer’s guidelines. Nucleotide sequencing was carried out using the Sanger sequencing method (Life Technologies, Carlsbad, CA, USA) in an automated genetic analyzer (ABI 3500xL). Nucleotide sequences were analyzed using Chromas 2.23 (Technelysium Pty Ltd., Queensland, Australia; available at http://www.technelysium.com.au/chromas.html.) [39].

For phylogenetic analysis, sequences were deposited from the NCBI/GenBank database using the BLAST search tool available online (https://www.nlm.nih.gov). Sequences of more than 98% similarity were selected and aligned to construct a phylogenetic tree using MEGA 7 software version 7.0. The maximum-likelihood method established on the Kimura 2-parameter model was used to infer the evolutionary history [40]; 1000 bootstraps were used to reconstruct the tree and missing data were discarded. At branch nodes, all bootstrap values were placed and the number of changes per nucleotide position was shown at the scale bar.

### 2.10. Statistical Analysis

Field and laboratory data were entered in a Microsoft Excel spreadsheet (Microsoft Corp., Washington, DC, USA). The data were cleaned, coded, recoded and checked for integrity before being exported to STATA/IC- 13 (STATA Corp LLC, Lakeway Drive, TX, USA) for epidemiological analysis. Descriptive analysis was performed to determine the overall individual level of AI seroprevalence and viral RNA prevalence based on c-ELISA and rRT-PCR tests, respectively. AI seroprevalence and viral RNA prevalence based on different variables were assessed, as well expressed as a percentage, frequency number and 95% confidence interval (CI).

Univariate analysis was done for different factors related to farmer’s demographic status and management practices in the study location. Factors of farmer’s education, study sites, year, duck types and contact types were initially assessed by the Chi-square test to identify the univariable association between AI serological prevalence and viral RNA prevalence. After identifying significant factors (*p* ≤ 0.2) in univariate analysis, significant associated factors were forwarded to the multivariate logistic regression model. The multivariate logistic model, however, did not fit well when tested through LRT (Likelihood Ratio Test) because co-linearity was investigated between independent variables by the Chi-square test and identified as problematic for a good fit of the model. Therefore, only univariable logistic regression was performed to check the strength between different categories of potential factors and AI serological and viral RNA prevalence. In comparison, the outputs of the univariable logistic regression were expressed in Odds Ratio (OR), *p*-value and 95% CI.

## 3. Results

### 3.1. Free-Range and Household Ducks Samples

In the present study, a total number of 2841 duck samples were collected and tested. During the three consecutive winters of 2015–2017, specifically in the month of December of each year, we sampled a total of 947 ducks. Three samples (blood sample, oropharyngeal and cloacal swabs) were collected from each duck. The numbers of sampled ducks were 312 for free-range ducks and 635 for household ducks, respectively. The free-range ducks (*n* = 312) were collected from 60 flocks (five ducks per flock) and additional samples (*n* = 12) were collected from two flocks (six ducks per flock). For free-range ducks, in 2015, we sampled 316 ducks and we collected a total number of 948 samples (316 samples each of blood, C and OP). In 2016, 314 ducks were sampled and number of 942 samples (314 samples each of blood, C and OP). In 2017, 317 ducks were sampled and a total number of 951 samples (317 samples each of blood, C and OP) were collected.

### 3.2. Avian Influenza Serological and Viral RNA Prevalence in Free-Range and Household Ducks

The overall AI seroprevalence and viral RNA prevalence in ducks were 63.8% (95% CI: 60.6–66.8; *n* = 947) and 10.7% (8.8–12.8; *n* = 947), respectively. In case of free-range duck flocks, AI seroprevalence was 90.3% (80.1–96.4; *n* = 62) and AI viral RNA prevalence was 12.9% (5.7–23.9; *n* = 62). In contrast, the AI seroprevalence and viral RNA prevalence in household duck flocks were 66.3% (62.5–69.9; *n* = 635) and 11.2 (8.8–13.9; *n* = 635), respectively (Table 1).

**Figure 2:**
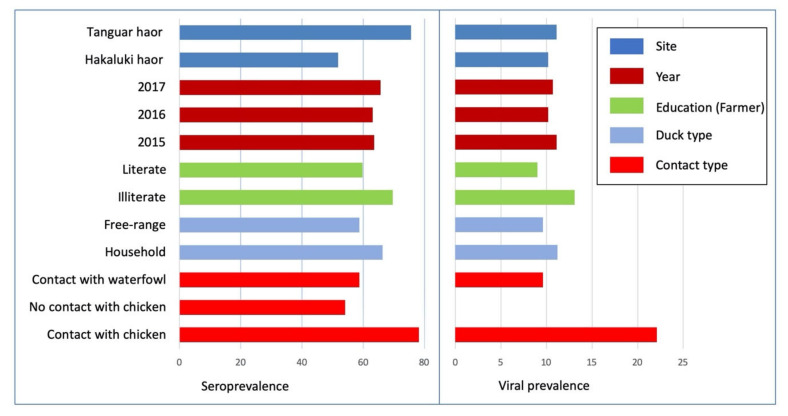
Distribution of AI serological prevalence and viral RNA prevalence in ducks, by different factors and categories in the present study.

AI serological and viral RNA prevalence rates were determined for duck flocks owned by farmers with varying levels of education. AI seroprevalence (69.6%, 95% CI: 64.8–74.2) was higher in ducks of the illiterate farmers group than in ducks of the literate farmers (59.8%, 95% CI: 55.6–63.9)) and similarly, in the same group, AI viral RNA prevalence was higher in illiterate farmers group (13.1%, 9.8–16.9) than AI viral prevalence in the literate farmer group (9.0%, 95% CI: 6.8–11.7) (Figure 2).

The seroprevalence rate in Tanguar was higher (75.6%; 95% CI: 71.5–79.4) than that in Hakaluki (51.8%, 47.2–56.4) and the viral RNA prevalence rates were lower (10.2%, 95% CI: 7.6–13.3) in Hakaluki haor than the Tanguar haor (11.1%, 8.5–14.3) (Figure 2).

AI seroprevalence was 58.7% (95% CI: 52.9–64.2) in free-range ducks and 66.3% (62.5–69.9) in household ducks. On the contrary, viral RNA prevalence was 9.6% (6.5–13.4) in free-range ducks and 11.2% (8.8–13.9) in household duck (Figure 2).

Both AI seroprevalence (78.2%, 95% CI: 73.3–82.6) and viral RNA prevalence (22.1%, 17.7–27) were higher in ducks which had contact with chickens than in ducks with no contact with chicken. There was no viral RNA detected in ducks which had no contact with chickens (0%,95% CI: 0.00–0.01). On the contrary, ducks in contact with waterfowl showed AI seroprevalence of 58.7% (52.9–64.2) and viral RNA prevalence of 9.6% (6.6–13.4), respectively (Figure 2).

### 3.3. Risk Factor Analysis of Individual-Level AI Serological and Viral RNA Prevalence

AI seroprevalence was significantly higher in household ducks of Tanguar haor (*p* < 0.001) than Hakaluki haor. In case of the rearing system, AI seroprevalence was considerably higher in ducks of illiterate farmers (*p* = 0.002) than literate farmers. AI seroprevalence was also higher in ducks in contact with chickens (*p* < 0.001) than waterfowl. AI viral RNA prevalence was significantly higher in the ducks of illiterate farmers (*p* = 0.047) and in ducks that were in contact with both chickens and ducks (*p* < 0.001) (Table 2).

### 3.4. Risk Factor Analysis of AI Serological and Viral RNA Prevalence in Free-Range Duck Flocks and Household Duck Flocks

There was no significant variation in AI seroprevalence in free-range duck flocks. On the other hand, flock level AI seroprevalence was significantly higher in household duck flocks in Tanguar haor (*p* < 0.001), those owned by illiterate farmers (*p* = 0.001) and those in contact with chicken (*p* < 0.001). AI viral RNA prevalence was significantly higher in free-range duck flocks reared by illiterate farmers (*p* = 0.006). Moreover, AI viral RNA prevalence was significantly higher in household duck flocks in contact with chicken (*p* < 001) (Table 3).

### 3.5. Univariable Logistic Regression of the Effect of Risk Factors on AI Seroprevalence in Ducks

**Figure 3:**
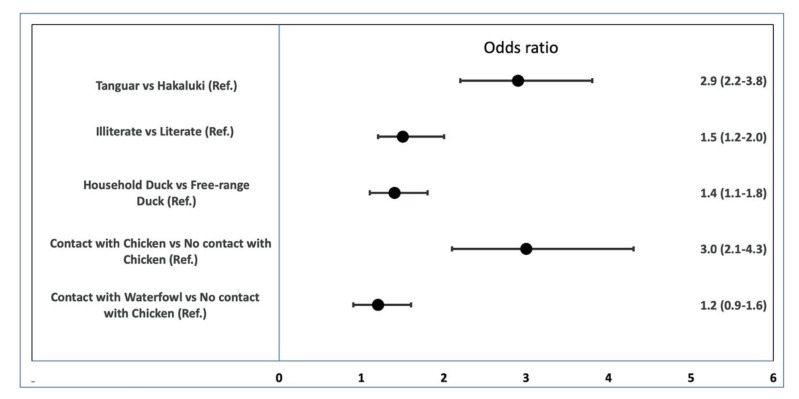
Representation of the odds ratios (OR) and confidence interval of AI serological prevalence by associated factors. The Odd ratios were found to be significantly associated with different factors; points denote the odds ratio and whiskers denote the 95% confidence interval.

The odds of AI seroprevalence were 1.5 time (95% CI: 1.2–2.0) higher (*p* = 0.002) in illiterate farmers than in literate farmers. In terms of location, the likelihood of AI seroprevalence was 2.9 (2.2–3.8) times higher in Tanguar than in Hakaluki (*p* < 0.001). The odds ratio was 1.4 (1.1–1.8) times higher in ducks reared in households than in the free-range ducks. Ducks in contact with chickens had 3.0 times higher odds (2.1–4.3) of having AI seroprevalence (*p* < 0.001) than did ducks which had no contact with chickens (Figure 3).

### 3.6. Univariable Logistic Regression of the Effect of Risk Factors on AI Viral RNA Prevalence in Ducks

**Figure 4:**
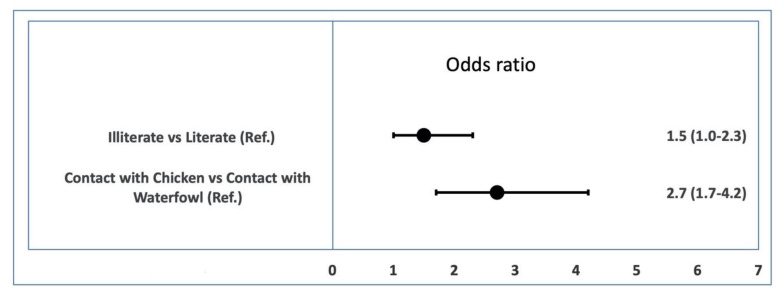
Representation of the odds ratios and confidence interval of AI viral RNA prevalence by associated factors. The odd ratios were found to be significantly associated with different factors; points denote the odds ratio and whiskers denote the 95% confidence interval.

The odds ratio was 1.5 (95% CI: 1.0–2.3) in farmers who were illiterate compared to that of the literate group (*p* = 0.048). Following adjustment in the category of risk factor ‘contact type,’ the odds ratio of AI prevalence in the category ‘chicken and duck contact’ was 2.7 (1.7–4.2) compared with the category that were only in contact with duck (*p* < 0.001) (Figure 4).

### 3.7. Phylogenetic Analysis

**Figure 5:**
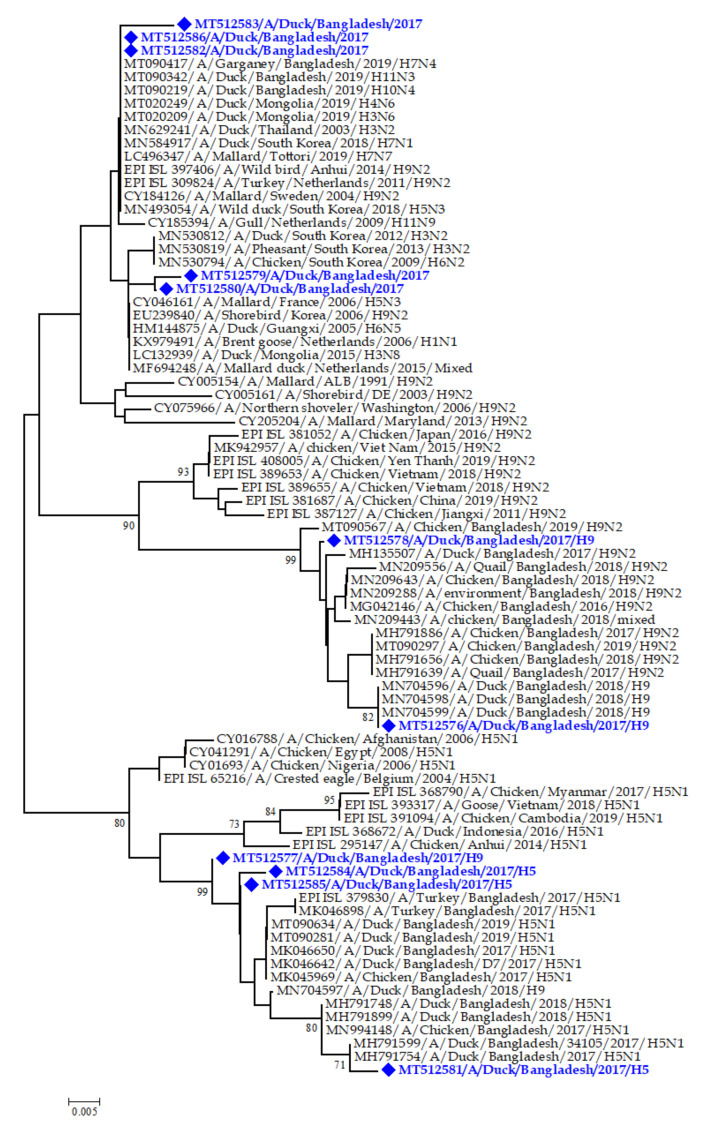
Phylogenetic tree of the M gene sequences detected in ducks in the wetlands of Hakaluki and Tanguar haor in Bangladesh in 2015–2017, generated by neighbor-joining method in MEGA 7. Bootstrap values ≥70 are indicated at the branches. Blue diamonds (♦) and the taxon name in blue colored text indicated the M-gene sequences identified during the study period.

A total of 11 M gene positive samples were partially sequenced (243 bp). The M gene partial sequences were deposited in NCBI/GenBank under the accession numbers MT512576, MT512577, MT512578, MT512579, MT512580, MT512581, MT512582, MT512583, MT512584, MT512585 and MT512586. The phylogenetic analysis of the M gene partial sequences of IAVs in the present study suggested that these sequences were similar to sequences of IAVs isolated from wild ducks, domestic ducks, chickens and shorebirds reported from South Korea and domestic ducks from Thailand, Mongolia and Netherlands (Figure 5). Three M gene sequences (Accession numbers MN704596, MN704598 and MN704599) were clustered tightly with the sequence of influenza virus A/H9N2 subtype reported from a duck in Bangladesh in 2018. However, another two (Accession numbers MH791599 and MH791754) sequences were similar to influenza virus A/H5N1 subtype reported from duck in Bangladesh in 2017. Moreover, another five sequences have close similarity with M gene of multiple low pathogenic influenza virus A/H3N2 subtype in South Korea and influenza virus A/H7N4 subtype in Garganey waterfowl in wetland in Bangladesh (Figure 5).

## 4. Discussion

Avian influenza epidemiological studies in Bangladesh were previously conducted in different chicken farming systems and live bird markets without much emphasis on duck farming and AI monitoring [25]. The present study provides the serological and molecular evidence of IAV infections among ducks in the wetland areas in Bangladesh. High AI seroprevalence (63.8%) in ducks at an individual level was recorded in the present study, which is similar to a previous study (55.6%) conducted in the northeastern and western parts of Bangladesh [41]. Variable individual level of AI seroprevalence was previously reported to be 15–90.2% in Bangladesh [8,9,13,14,42]. Complex production systems involving household ducks and rice production are likely to be important epidemiological factors for the maintenance and spread of IAVs, which might explain the existing high AI seroprevalence in the study sites [43].

The overall individual level of AI viral prevalence in the present study was estimated at 10.7%. Other studies conducted on household ducks in Bangladesh reported prevalence rates which ranged from 0.05% [9] and 4.4% [44] to 22% [8] and 89% [15]. The later research was based on clinically sick ducks likely explaining higher values. H3, H7 and H15 avian influenza virus subtypes were previously reported in ducks of the haor areas [45].

The higher AI seroprevalence and viral RNA prevalence rates indicate that household ducks might be a source of infection for poultry in haor areas of Bangladesh. However, whether ducks became infected from chickens as a result of spillback is unknown and require further investigation. In terms of ducks having contact with household ducks, the absence of viral RNA might be due to the absence of IAVs circulation at the time of surveillance, resulted from a short shedding period of IAVs in ducks [46]. The AI seroprevalence was 58.5% in ducks having contact with wild waterfowl and viral RNA prevalence was 9.6%. Previous studies have shown that free-range ducks contact with wild waterfowl was affected by IAVs during winter [27]. The correlations between the risk of HPAI outbreaks and the presence of domestic waterfowl was established in previous studies [47]. In contrast, the association of higher AI prevalence with the migration site was also known [48].

From 2015 to 2017, no significant differences in AI seroprevalence or AI viral RNA prevalence were identified. During this period, Bangladesh had reported a total of 8 H5N1 outbreaks in poultry. Winter is considered favorable season for AI occurrence and higher presence of IAVs was recorded in Bangladesh and Southeast Asia in winter months [49]. The long-term persistence of IAVs in the environment, cold water (up to 207 days at 17 °C) and a low relative humidity of 20–35% may explain the higher AI prevalence in winter [50]. It was previously reported that the existence of migratory waterfowl in Bangladesh during winter months plays an active role in transmission and shedding of IAVs into other domestic avian hosts [48,51]. In contrast, the persistence of IAVs in the migratory waterfowl stopover sites may facilitate indirect transmission of IAVs to household ducks and chickens [52].

According to the spatial distribution, Tanguar haor was identified as a risk factor of higher odds of IAV than Hakaluki haor. This finding does not support a previous study where Hakaluki haor was reported to have more IAV prevalence than Tanguar haor [8]. This might be explained by difference in the duck type, where semi-scavenging household ducks were tested [8]. In contrast, the findings of the mentioned study were not established by analyzing the odds, which was a statistical limitation. Every year, around 60 species of migratory wild birds come to Tanguar haor for their winter habitat [53]. The haor water body is shared by a large number of migratory waterfowl, resident birds and free-range ducks and this situation amplifies the risk of IAVs circulation [6,7,54]. The present study reported a significant amount (OR 3) of seropositive ducks having contact with household chickens than those with no contact with chicken. Outbreak data have confirmed the contribution of household ducks to HPAI outbreaks in poultry in Southeast Asia as previously reported [55].

The odds of ducks being seropositive were higher in household ducks (OR 1.4) than in free-range ducks in the present study. In case of viral RNA prevalence, no such strength had been found between the rearing system and AI occurrence. The updated view regarding this is that no rearing system can be blamed more for spreading the infectious disease, while incremental biosecurity measures in all kinds of rearing systems are recommended [56]. Based on the farmers’ demography, the odds of AI ducks being seropositive and viral RNA positive were higher among ducks reared by illiterate farmers than among ducks reared by literate farmers (OR 1.5 for both). The connection between the farmer’s educational levels and higher AI prevalence can be deduced as a disease prevention depending on the farmer’s precautionary behavior, such as biosecurity implementation, perception of risk and loss of profit [56]. Studies had shown a significant association between farmer’s education with AI and zoonotic awareness, whereas knowledgeable farmers tend to take different precautionary measures to control infectious diseases [57]. In this study, AI seroprevalence and viral RNA prevalence were higher in free-range duck flocks than that of household duck flocks, which is consistent with previous studies [27]. It is likely that the frequent movement of free-range ducks in the wetlands provides more exposure and interaction with wild migratory birds and other wild waterfowl leading to higher infection rates.

Phylogenetic analysis revealed that the five M gene partial sequences of IAVs obtained from Tanguar haor and Hakaluki haor were similar to M gene sequence from LPAI subtypes detected in waterfowl in Bangladesh, South Korea, Thailand and Mongolia [58]. Among them, three sequences showed similarities with sequences from ducks and Garganey waterfowl in Bangladesh as previously reported GenBank accession number (MT090342 and MT09417), which might be due to the substantial interaction between free-range ducks and wild migratory birds in the distinct wetland ecosystem. The remaining 6 out of 11 M gene sequences has close genetic relatedness to M gene of IAV H5 and H9 subtypes detected in the duck and chicken samples in Bangladesh between 2017 to 2019 [12,59,60], indicating that those clades of IAVs were circulating throughout the poultry species in Bangladesh.

The present study had some limitations. These include the fact that IAVs were detected using M gene specific rRT-PCR and no subtype determination was performed. In addition, only M gene sequence data were determined due to limited resources. We are currently characterizing the hemagglutinin (HA) and neuraminidase (NA) genes to understand the actual genetic diversity of IAVs circulating in free-range ducks in Bangladesh.

## 5. Conclusions

The findings of the present study showed high AI seroprevalence and viral RNA prevalence in duck population of Hakaluki and Tanguar haors. Tanguar haor was identified with significantly higher seropositive ducks, possibly due to the density and types of migratory waterfowl. A farmer’s low educational status, household duck rearing system and contact of ducks with household chickens and migratory waterfowl were found to be associated with higher AI prevalence. The outcomes of the current study will help model future IAV infection to predict the spread of IAVs in major wetlands in Bangladesh. The findings of the present study recommended the implementation of risk communication for farmers and other stakeholders and strengthening surveillance and control strategies in human and domestic poultry health sectors using the One Health approach.

## Figures and Tables

**Table 1:** Frequency distribution of AI serological prevalence and viral RNA prevalence in the study areas in Bangladesh between 2015 and 2017.

Type of Sample	Test (*n*)	Positive Number (*n*)	Percentage (%)	95% CI
Total ducks	c-ELISA (947)	604	63.8	60.6–66.8
	rRT-PCR (947)	101	10.7	8.8–12.8
Free-range duck flocks	c-ELISA (62) *	56	90.3	80.1–96.4
	rRT-PCR (62) *	8	12.9	5.7–23.9
Household duck flocks	c-ELISA (635)	421	66.3	62.5–69.9
	rRT-PCR (635)	71	11.2	8.8–13.9

CI: Confidence Interval; *n*: total number of samples, *: the number of flocks (62).

**Table 2:** Univariable association between different factors and AI serological prevalence and viral RNA prevalence.

Factor	Category	c-ELISA			rRT-PCR		
		Number of Positive Samples (%)	95% CI	*p*	Number of Positive Samples (%)	95% CI	*p*
**Wetlands**	Hakaluki haor (471)	244 (51.8)	47.2–56.4	<0.001	48 (10.2)	7.6–13.3	0.638
	Tanguar haor (476)	360 (75.6)	71.5–79.4		53 (11.1)	8.5–14.3	
**Year**	2015 (316)	201 (63.6)	58.0–68.9	0.912	35 (11.1)	7.8–15.1	0.936
	2016 (314)	198 (63.1)	57.5–68.4		32 (10.2)	7.1–14.1	
	2017 (317)	205 (65.7)	59.1–69.9		34 (10.7)	7.5–14.6	
**Education (Farmer)**	Illiterate (382)	266 (69.6)	64.8–74.2	0.002	50 (13.1)	9.8–16.9	0.047
	Literate (565)	338 (59.8)	55.6–63.9		51 (9.0)	6.8–11.7	
**Duck type**	Household (635)	421 (66.3)	62.5–69.9	0.021	71 (11.2)	8.8–13.9	0.463
	Free-range (312)	183 (58.7)	52.9–64.2		30 (9.6)	6.5–13.4	
**Contact type**	Contact with Chicken (321)	251 (78.2)	73.3–82.6	<0.001	71 (22.1)	17.7–27.1	<0.001
	No contact with Chicken (314)	170 (54.1)	48.5–59.7		0(0)	0–1.16	
	Contact with Waterfowl (312)	183 (58.7)	52.9–64.2		30 (9.6)	6.6–13.4	

**Table 3:** Pairwise comparison of AI serological prevalence and viral RNA prevalence between free-range and household duck flocks.

Factor	Category	c-ELISA				rRT-PCR			
		Free-Range Duck Flocks		Household Duck Flocks		Free-Range Duck Flocks		Household Duck Flocks	
		Number of Positives (%)	*p*	Number of Positives (%)	*p*	Number of Positives (%)	*p*	Number of Positives (%)	*p*
**Wetlands**	Hakaluki haor	28 (90.3)	1.000	153 (48.4)	<0.001	3 (9.7)	0.449	39 (12.3)	0.356
	Tanguar haor	28 (90.3)		268 (84.0)		5 (16.1)		32 (10.0)	
**Year**	2015	19 (90.5)	0.998	140 (66.4)	0.971	3 (14.3)	0.849	25 (11.9)	0.922
	2016	19 (90.5)		136 (65.7)		2 (9.5)		22 (10.6)	
	2017	18 (90)		145 (66.8)		3 (15)		24 (11.1)	
**Education (Farmer)**	Illiterate	18 (90)	0.953	201 (73.6)	0.001	6 (30)	0.006	23 (8.4)	0.056
	Literate	38 (90.5)		220 (60.8)		2 (4.8)		48 (13.3)	
**Contact type**	Contact with Chicken	-	-	251 (78.2)	<0.001	-	-	71 (22.1)	<0.001
	No contact with Chicken	-	-	170 (54.1)		-	-	0	
	<10	-	-	392 (66.2)	0.870	-	-	7 (16.3)	0.272
**Farm/Flock size**	≥10	-	-	29 (67.4)		-	-	64 (10.8)	
	<500	38 (92.7)	0.380	-	-	5 (12.2)	0.816	-	-
	≥500	18 (85.7)		-	-	3 (14.3)		-	-

## Supplementary Materials

The following are available online at https://www.mdpi.com/2076-0817/9/11/953/s1, Table S1: metadata of domestic ducks samples during avian influenza surveillance in 2015–2017 in Bangladesh.

## References

[1] Capua I., Alexander D.J. (2007). Avian influenza infections in birds—A moving target. Influenza Other Respir. Viruses.

[2] Riedel S. (2006). Crossing the species barrier: The threat of an avian influenza pandemic. Baylor University Medical Center Proceedings.

[3] Marchenko V.Y., Alekseev A., Sharshov K., Petrov V., Silko N., Susloparov I., Tserennorov D., Otgonbaatar D., Savchenko I., Shestopalov A. (2012). Ecology of influenza virus in wild bird populations in Central Asia. Avian Dis..

[4] Gu M., Liu W., Cao Y., Peng D., Wang X., Wan H., Zhao G., Xu Q., Zhang W., Li Y. (2011). Novel reassortant highly pathogenic avian influenza (H5N5) viruses in domestic ducks, China. Emerg. Infect. Dis..

[5] Kim H.-R., Park C.-K., Oem J.-K., Bae Y.-C., Choi J.-G., Lee O.-S., Lee Y.-J. (2010). Characterization of H5N2 influenza viruses isolated in South Korea and their influence on the emergence of a novel H9N2 influenza virus. J. Gen. Virol..

[6] Cappelle J., Zhao D., Gilbert M., Nelson M.I., Newman S.H., Takekawa J.Y., Gaidet N., Prosser D.J., Liu Y., Li P. (2014). Risks of avian influenza transmission in areas of intensive free-ranging duck production with wild waterfowl. EcoHealth.

[7] Hassan M.M., Hoque M.A., Ujvari B., Klaassen M. (2018). Live bird markets in Bangladesh as a potentially important source for Avian Influenza Virus transmission. Prev. Vet. Med..

[8] Khatun A., Giasuddin M., Islam K.M., Khanom S., Samad M.A., Islam M.R., Noor M., Bhuiyan J.U., Kim W.-I., Eo S.K. (2013). Surveillance of avian influenza virus type A in semi-scavenging ducks in Bangladesh. BMC Vet. Res..

[9] Sarker R.D., Giasuddin M., Chowdhury E.H., Islam M.R. (2017). Serological and virological surveillance of avian influenza virus in domestic ducks of the north-east region of Bangladesh. BMC Vet. Res..

[10] Parvin R., Kamal A.H., Haque M.E., Chowdhury E.H., Giasuddin M., Islam M.R., Vahlenkamp T.W. (2014). Genetic characterization of highly pathogenic H5N1 avian influenza virus from live migratory birds in Bangladesh. Virus Genes.

[11] Biswas P.K., Christensen J.P., Ahmed S.S., Barua H., Das A., Rahman M.H., Giasuddin M., Hannan A.S., Habib M.A., Ahad A. (2008). Avian influenza outbreaks in chickens, Bangladesh. Emerg. Infect. Dis..

[12] Gerloff N.A., Khan S.U., Balish A., Shanta I.S., Simpson N., Berman L., Haider N., Poh M.K., Islam A., Gurley E. (2014). Multiple reassortment events among highly pathogenic avian influenza A (H5N1) viruses detected in Bangladesh. Virology.

[13] Hassan M., Das B., Mahmud M., Amin M., Yousuf M., Jaber M., Belal S., Hasan M., Hossen A., Karim M. (2015). Seroprevalence and detection of avian influenza type A in ducks at Nikli and Bajitpur upazila of Bangladesh. Bangladesh J. Vet. Med..

[14] Hassan M.M., El Zowalaty M.E., Islam A., Khan S.A., Rahman M.K., Järhult J.D., Hoque M.A. (2020). Prevalence and Diversity of Avian Influenza Virus Hemagglutinin Sero-Subtypes in Poultry and Wild Birds in Bangladesh. Vet. Sci..

[15] Haider N., Sturm-Ramirez K., Khan S., Rahman M., Sarkar S., Poh M., Shivaprasad H., Kalam M., Paul S., Karmakar P. (2017). Unusually high mortality in waterfowl caused by highly pathogenic avian influenza A (H5N1) in Bangladesh. Transbound. Emerg. Dis..

[16] Turner J.C., Feeroz M.M., Hasan M.K., Akhtar S., Walker D., Seiler P., Barman S., Franks J., Jones-Engel L., McKenzie P. (2017). Insight into live bird markets of Bangladesh: An overview of the dynamics of transmission of H5N1 and H9N2 avian influenza viruses. Emerg. Microbes Infect..

[17] Biswas P.K., Christensen J.P., Ahmed S.S., Das A., Rahman M.H., Barua H., Giasuddin M., Hannan A.S., Habib M.A., Debnath N.C. (2009). Risk for infection with highly pathogenic avian influenza virus (H5N1) in backyard chickens, Bangladesh. Emerg. Infect. Dis..

[18] Loth L., Gilbert M., Osmani M.G., Kalam A.M., Xiao X. (2010). Risk factors and clusters of highly pathogenic avian influenza H5N1 outbreaks in Bangladesh. Prev. Vet. Med..

[19] Ahmed S.S., Ersbøll A.K., Biswas P.K., Christensen J.P., Hannan A.S., Toft N. (2012). Ecological determinants of highly pathogenic avian influenza (H5N1) outbreaks in Bangladesh. PLoS ONE.

[20] Kamruzzaman M., Shaw R. (2018). Flood and sustainable agriculture in the Haor basin of Bangladesh: A review paper. Univers. J. Agric. Res..

[21] Jha B., Hossain M., Baishnab P., Mandal P., Islam M. (2015). Socio-economic status of duck farmers and duck farming in haor areas of Sylhet district in Bangladesh. Int. J. Nat. Sci..

[22] Khanum R., Al Mahadi M.S. (2015). Economic empowerment of haor women through duck farming in Bangladesh. Agriculturists.

[23] Chakraborty T.R. (2009). Management of haors, baors and beels in Bangladesh. Lessons Lake Basin Manag..

[24] Ghosh S., Haider N., Khan M. (2012). Status of Household Ducks and their Associated Factors under Scavenging System in a Southern Area of Bangladesh. Int. J. Nat. Sci..

[25] Hoque M., Skerratt L., Cook A., Khan S., Grace D., Alam M., Vidal-Diez A., Debnath N. (2011). Factors limiting the health of semi-scavenging ducks in Bangladesh. Trop. Anim. Health Prod..

[26] Hoque M.A., Hassan M.M., Haque E., Shaikat A.H., Khan S.A., Alim A., Skerratt L.F., Islam A., Tun H.M., Dissanayake R. (2014). A survey of gastro-intestinal parasitic infection in domestic and wild birds in Chittagong and Greater Sylhet, Bangladesh. Prev. Vet. Med..

[27] Sarkar S., Khan S.U., Mikolon A., Rahman M.Z., Abedin J., Zeidner N., Sturm-Ramirez K., Luby S.P. (2017). An epidemiological study of avian influenza A (H5) virus in nomadic ducks and their raising practices in northeastern Bangladesh, 2011–2012. Influenza Other Respir. Viruses.

[28] Wan X. (2012). Lessons from emergence of a/goose/guangdong/1996-like h5n1 highly pathogenic avian influenza viruses and recent influenza surveillance efforts in southern china. Zoonoses Public Health.

[29] World Health Organization (2020). Cumulative Number of Confirmed Human Cases for Avian Influenza A (H5N1) Reported to WHO, 2003–2020.

[30] Chakma D., Rushton J. (2008). Rapid Assessment on Socio-Economic Impact due to Highly Pathogenic Avian Influenza in Bangladesh.

[31] Hassan M.M., Hoque M.A., Debnath N.C., Yamage M., Klaassen M. (2017). Are poultry or wild birds the main reservoirs for avian influenza in Bangladesh?. Ecohealth.

[32] Choudhury G.A., Nishat A. (2005). Hydro-Meteorological Characteristics of Hakaluki Haor.

[33] Nowreen S., Murshed S.B., Islam A.S., Bhaskaran B., Hasan M.A. (2015). Changes of rainfall extremes around the haor basin areas of Bangladesh using multi-member ensemble RCM. Theor. Appl. Climatol..

[34] Druce J., Garcia K., Tran T., Papadakis G., Birch C. (2012). Evaluation of swabs, transport media and specimen transport conditions for optimal detection of viruses by PCR. J. Clin. Microbiol..

[35] Selleck P. (2007). Influenza A Virus: A Competitive ELISA for the Detection of Antibodies to Influenza a Viruses in Equine Sera, Equine Influenza c-ELISA Protocol.

[36] Spackman E., Senne D.A., Myers T., Bulaga L.L., Garber L.P., Perdue M.L., Lohman K., Daum L.T., Suarez D.L. (2002). Development of a real-time reverse transcriptase PCR assay for type A influenza virus and the avian H5 and H7 hemagglutinin subtypes. J. Clin. Microbiol..

[37] Monne I., Ormelli S., Salviato A., De Battisti C., Bettini F., Salomoni A., Drago A., Zecchin B., Capua I., Cattoli G. (2008). Development and validation of a one-step real-time PCR assay for simultaneous detection of subtype H5, H7 and H9 avian influenza viruses. J. Clin. Microbiol..

[38] Anthony S., Leger J.S., Pugliares K., Ip H.S., Chan J., Carpenter Z., Navarrete-Macias I., Sanchez-Leon M., Saliki J., Pedersen J. (2012). Emergence of fatal avian influenza in New England harbor seals. MBio.

[39] Puthavathana P., Auewarakul P., Charoenying P.C., Sangsiriwut K., Pooruk P., Boonnak K., Khanyok R., Thawachsupa P., Kijphati R., Sawanpanyalert P. (2005). Molecular characterization of the complete genome of human influenza H5N1 virus isolates from Thailand. J. Gen. Virol..

[40] Kimura D.K. (1980). Likelihood methods for the von Bertalanffy growth curve. Fish. Bull..

[41] Ansari W.K., Parvej M.S., El Zowalaty M.E., Jackson S., Bustin S.A., Ibrahim A.K., El Zowalaty A.E., Rahman M.T., Zhang H., Khan M.F.R. (2016). Surveillance, epidemiological and virological detection of highly pathogenic H5N1 avian influenza viruses in duck and poultry from Bangladesh. Vet. Microbiol..

[42] Hassan M.M., El Zowalaty M.E., Islam A., Rahman M.M., Chowdhury M.N., Nine H.S., Rahman M.K., Järhult J.D., Hoque M.A. (2020). Serological Evidence of Avian Influenza in Captive Wild Birds in a Zoo and Two Safari Parks in Bangladesh. Vet. Sci..

[43] Pfeiffer D.U., Minh P.Q., Martin V., Epprecht M., Otte M.J. (2007). An analysis of the spatial and temporal patterns of highly pathogenic avian influenza occurrence in Vietnam using national surveillance data. Vet. J..

[44] Khan S.U., Gurley E.S., Gerloff N., Rahman M.Z., Simpson N., Rahman M., Haider N., Chowdhury S., Balish A., Zaman R.U. (2018). Avian influenza surveillance in domestic waterfowl and environment of live bird markets in Bangladesh, 2007–2012. Sci. Rep..

[45] El-Shesheny R., Feeroz M.M., Krauss S., Vogel P., McKenzie P., Webby R.J., Webster R.G. (2018). Replication and pathogenic potential of influenza A virus subtypes H3, H7 and H15 from free-range ducks in Bangladesh in mammals. Emerg. Microbes Infect..

[46] Latorre-Margalef N., Gunnarsson G., Munster V.J., Fouchier R.A., Osterhaus A.D., Elmberg J., Olsen B., Wallensten A., Haemig P.D., Fransson T. (2009). Effects of influenza A virus infection on migrating mallard ducks. Proc. R. Soc. B Biol. Sci..

[47] Gilbert M., Newman S.H., Takekawa J.Y., Loth L., Biradar C., Prosser D.J., Balachandran S., Rao M.V.S., Mundkur T., Yan B. (2010). Flying over an infected landscape: Distribution of highly pathogenic avian influenza H5N1 risk in South Asia and satellite tracking of wild waterfowl. Ecohealth.

[48] Ahmed S.S., Ersbøll A.K., Biswas P.K., Christensen J.P., Toft N. (2011). Spatio-temporal magnitude and direction of highly pathogenic avian influenza (H5N1) outbreaks in Bangladesh. PLoS ONE.

[49] Giasuddin M., Haque M., Kamal A., Islam M., Jahangir A., Chowdhury E., Taimur M., Rahman M.H. (2012). Outbreak evaluation of highly pathogenic avian influenza in Bangladesh. Bangladesh J. Livest. Res..

[50] Lowen A.C., Mubareka S., Steel J., Palese P. (2007). Influenza virus transmission is dependent on relative humidity and temperature. PLoS Pathog..

[51] Barman S., Marinova-Petkova A., Hasan M.K., Akhtar S., El-Shesheny R., Turner J.C., Franks J., Walker D., Seiler J., Friedman K. (2017). Role of domestic ducks in the emergence of a new genotype of highly pathogenic H5N1 avian influenza A viruses in Bangladesh. Emerg. Microbes Infect..

[52] Lickfett T.M., Clark E., Gehring T.M., Alm E.W. (2018). Detection of Influenza A viruses at migratory bird stopover sites in Michigan, USA. Infect. Ecol. Epidemiol..

[53] Alam A., Chowdhury M., Sobhan I. (2012). Biodiversity of Tanguar Haor: A Ramsar Site of Bangladesh. Wildl. IUCN Bangladesh Dhaka..

[54] Hassan M.M. (2017). Who Is the Culprit: Ecology and Epidemiology of Avian Influenza at the Wildlife-Poultry Interface in Bangladesh. Ph.D. Thesis.

[55] Gilbert M., Chaitaweesub P., Parakamawongsa T., Premashthira S., Tiensin T., Kalpravidh W., Wagner H., Slingenbergh J. (2006). Free-grazing ducks and highly pathogenic avian influenza, Thailand. Emerg. Infect. Dis..

[56] Conan A., Goutard F.L., Sorn S., Vong S. (2012). Biosecurity measures for backyard poultry in developing countries: A systematic review. BMC Vet. Res..

[57] Sultana R., Rimi N.A., Azad S., Islam M.S., Khan M.S.U., Gurley E.S., Nahar N., Luby S.P. (2012). Bangladeshi backyard poultry raisers’ perceptions and practices related to zoonotic transmission of avian influenza. J. Infect. Dev. Ctries..

[58] Lee C.-W., Suarez D.L., Tumpey T.M., Sung H.-W., Kwon Y.-K., Lee Y.-J., Choi J.-G., Joh S.-J., Kim M.-C., Lee E.-K. (2005). Characterization of highly pathogenic H5N1 avian influenza A viruses isolated from South Korea. J. Virol..

[59] Shanmuganatham K., Feeroz M.M., Jones-Engel L., Walker D., Alam S., Hasan M., McKenzie P., Krauss S., Webby R.J., Webster R.G. (2014). Genesis of avian influenza H9N2 in Bangladesh. Emerg. Microbes Infect..

[60] Hoque M.A., Tun H.M., Hassan M.M., Khan S.A., Islam S.A., Islam M.N., Giasuddin M., Osmani T.M.G., Islam A., Thornton R.N. (2013). Molecular epidemiology of influenza A (H5N1) viruses, Bangladesh, 2007–2011. Prev. Vet. Med..

